# A Novel Ultra-Low Loss Rectangle-Based Porous-Core PCF for Efficient THz Waveguidance: Design and Numerical Analysis

**DOI:** 10.3390/s20226500

**Published:** 2020-11-14

**Authors:** Abdullah Al-Mamun Bulbul, Farjana Imam, Md. Abdul Awal, M. A. Parvez Mahmud

**Affiliations:** 1 Department of Electronics and Telecommunication Engineering, Bangabandhu Sheikh Mujibur Rahman Science and Technology University, Gopalganj-8100, Bangladesh; farjanabsmrstu@gmail.com; 2 Electronics and Communication Engineering Discipline, Khulna University, Khulna 9208, Bangladesh; m.awal@ece.ku.ac.bd; 3 School of Engineering, Deakin University, Geelong, VIC 3216, Australia; m.a.mahmud@deakin.edu.au

**Keywords:** confinement loss, dispersion, effective material loss, PCF, power fraction, THz waveguide

## Abstract

A novel, rectangle-based, porous-core photonic crystal fiber (PCF) has been modeled for the efficient propagation of a THz wave. The performance of the anticipated model has been assessed using the finite element method (FEM) in the range of 0.5–1.5 THz. Both the fiber core and cladding are modeled with rectangular air holes. Numerical analysis for this model reveals that the model has a lower amount of dispersion of about 0.3251 ps/THz/cm at 1.3 THz. Compared to the other THz waveguides, the model offers an ultra-lower effective material loss of 0.0039 cm^−1^ at the same frequency. The confinement loss is also lower for this model. Moreover, this model has a high-power fraction of about 64.90% at the core in the *x*-polarization mode. However, the effective area, birefringence, and numerical aperture have also been evaluated for this model. Maintenance of standard values for all the optical parameters suggests that the proposed PCF can efficiently be applied in multichannel communication and several domains of the THz technology.

## 1. Introduction

In the past few years, the terahertz (THz) wave radiation, which is also known as submillimeter-wave, has been potentially exercised in different applications like biosensing [1], medical imaging [2], astronomy [3], security [4], and communication [5,6]. THz radiation cannot go through metal or water, and it faces difficulty when it passes through fog and clouds. However, the non-ionizing feature makes them useful in other fields like medical imaging [4].

Although researchers have already achieved noteworthy success in the generation [7] and detection [8] of terahertz (THz) radiation, the propagation of this wave is still challenging. As the THz source is commercially available now, an efficient, low loss, and dispersion free THz waveguide is a burning need for the proper guidance of this wave. To realize these prerequisites, several types of the waveguide have been designed, such as metal wires [9], plastic fiber [10], dielectric tubes [11], hollow-core fiber [12], polymer Bragg fiber [13], and solid core fiber [14] for instance, the circular rod waveguide [15,16]. However, these waveguides suffer from numerous limitations, e.g., high material loss, higher bending loss, higher dispersion, and higher atmospheric loss.

Recently, researchers have focused their attention on the porous core-photonic crystal fiber (PCF) [17], where the designer can calibrate different parameters of the waveguide like pitch, core diameter, air-hole radius, and the operating frequency. Moreover, by varying the numerical factors of the waveguide parameters, it is feasible to realize standard values for the optical parameters, e.g., lower values for effective material loss (EML), dispersion, and confinement loss (CL), and higher values for the birefringence, and power fraction in the core region [13].

In current years, researchers have developed a variety of PCF models for multichannel communication and are continuously trying to attain further progress in this field. In 2015, Raonaqul et al. [18] introduced an asymmetric-core PCF model that exhibits ultra-high birefringence of about 0.075 at 1 THz. But this model demonstrates a higher dispersion of 0.5 ps/THz/cm. Rabiul et al. [19] proposed a polarization-maintaining spiral PCF-based THz waveguide. The model shows high birefringence and low confinement loss, but the values of other parameters such as dispersion and power fraction are poor. Another PCF model has been proposed in [20] where both core and cladding regions are asymmetric. They improved the EML, which is 0.08 cm^−1^, but they ignored the CL for their model. Also, they noticed a high dispersion of about 0.9 ± 0.26 ps/THz/cm. A diamond-core PCF for the THz transmission was introduced by Raonaqul et al. [21]. This model exhibits higher birefringence of the order 10^−2^ and lower EML of approximately 0.07 cm^−1^ at 0.7 THz, but the dispersion is very high for it. Zhiqing Wu et al. modelled an oligoporous-core PCF that shows high birefringence as well as low dispersion in a broad frequency range [22]. This PCF model offers a few improved outcomes from the previous models [18,19,20,21]. But the EML and CL for this model are high, and the power fraction was only 46%. In 2017, Kawsar Ahmed et al. [23] modelled a hexagonal porous core PCF for THz transmission. The EML and birefringence are the main focusing parameters for their model. However, they did not evaluate two essential parameters for communication purposes, namely the dispersion and CL. A new form of PCF model involving the slotted-core was proposed, which exhibits very low EML of about 0.0103–0.0145 cm^−1^ [24]. A square shape, porous-core THz waveguide was proposed by Jianfeng Luo et al. [25]. This model has lower dispersion. But it shows higher EML, higher CL, and a power fraction of about 46.9%. 

From the above discussion on different PCF-based THz waveguides, it is evident that researchers have done a lot of works for the efficient transmission of the THz wave employing various PCF models. Since research is still going on to achieve a novel waveguide with higher efficiency, there is still an opportunity to improve in different areas of the PCF model. The key contributions and salient features of this paper are as follows:

- A Zeonex-based PCF is modeled as a THz waveguide where both the core and cladding regions are modelled with rectangles.
- A noteworthy improvement is achieved which includes very low EML and low dispersion.
- The model also provides a lower CL and high core power fraction. The other parameters, such as birefringence, effective area, and numerical aperture are estimated for this model.

The paper is organized as follows: The design parameters for the proposed THz waveguide are discussed in Section 2 which is followed by the discussion on the fabrication feasibilities of this waveguide. Then, the mathematics behind the numerical analysis has been presented in Section 3. Finally, the results are analyzed in the subsequent section, followed by the presentation of an in-depth conclusion and future scopes of the proposed THz waveguide.

## 2. Design Methodology

**Figure 1:**
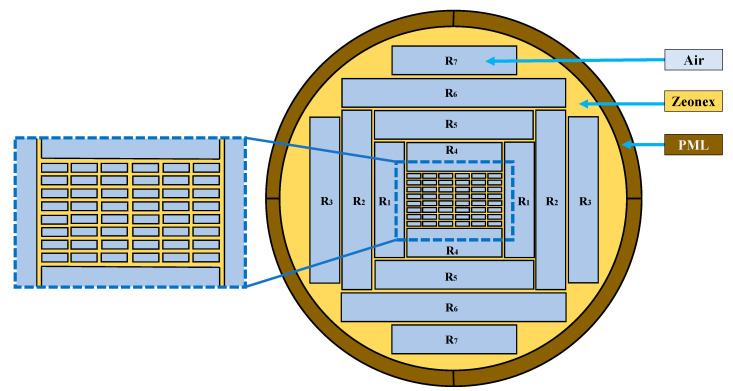
Cross-section of the proposed optimum PCF model.

The proposed PCF-based THz waveguide is modelled using the finite element method (FEM). The full vector FEM has homogenized the medium of the proposed PCF model [26]. Moreover, the effective material properties of the model have been evaluated using the full vector FEM. Figure 1 presents the x–y plan view of our proposed PCF model. The radius of the PCF is 1800 µm, including 140 µm Perfectly Matched Layer (PML). The core region consists of 48 small symmetric rectangles (hence the model is termed as 48 R). The width and height of a single rectangle are 145 µm and 53.75 µm correspondingly. The cladding region consists of 14 rectangles of different width and height. Among them, the width of R_1_, R_2_, and R_3_ are the same (300 µm), and the height of those are 1120 µm, 1740 µm, and 1208 µm individually. Again, the width of R_4_, R_5_, R_6_, and R_7_ are 920 µm,1540 µm, 2160 µm, and 1608 µm correspondingly, whereas the height of each of them are the same (300 µm). The value of strut is 10 µm for both core and cladding regions.

**Figure 2:**
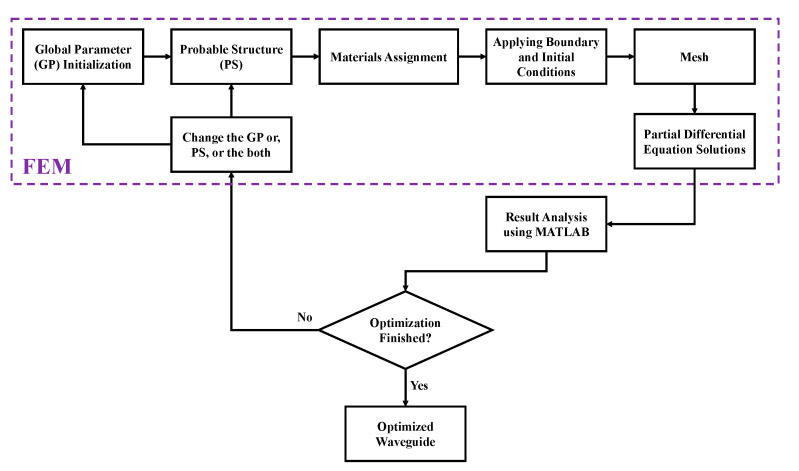
Sequential stages of the model design and analysis of this work.

Employing the trial and error method, we have designed the optimum model. However, we have compared this model with a few other models that result from tuning the number of rectangles and strut values of the core region. Firstly, by changing the number of rectangles in the core region, we have modelled two different PCF models, where the cladding region remains the same as the optimum model. One of the models has 24 rectangles (termed as 24 R) and a 10 µm strut value in both core and cladding regions. This model exhibits 84% porosity where the width and height of a single core rectangle are 300 µm and 53.75 µm individually. Another model has 80 rectangles (termed as 80 R) and the same strut value. This model exhibits 78% porosity, and the width and height of a single rectangle are 83 µm and 53.75 µm, respectively. Again, to evaluate the optimum porosity of the proposed model, we have changed the strut values of the core region, keeping the number of rectangles fixed as 48. Thereby, we have modelled two other PCF models with distinct porosity. The two chosen strut values are 7.5 µm and 12.5 µm for these two models. The model with a 7.5 µm strut has the highest porosity of about 86%. The width and height of a single rectangle are 147.08 µm and 55.94 µm respectively for this model. Again, the model with 12.5 µm strut value has the lowest porosity of about 77%. The width and height for a single rectangle in the core region of this model are 142.92 µm and 51.56 µm individually. Figure 2 depicts the stages involved in the design and analysis of the proposed model using FEM. The stages are presented consecutively. These steps are repeated multiple times until the typical values for the optical parameters are found.

The background material of our proposed PCF model is Zeonex (Cyclo Olefin Polymers), which maintains a static refractive index (RI) of 1.53 in the THz regime. Also, Zeonex exhibits numerous benefits such as low material dispersion in the THz band, high-temperature resistance, lower water absorption, high transparency, etc., compared to other existing polymers [27]. The tolerable temperature of Zeonex is 138 °C, which makes it more compatible with other high-temperature polymers. Also, it is more suitable for microstructure fiber fabrication than other existing polymers due to its high molecular weight, and it also ensures lower EML [27]. 

## 3. Fabrication Feasibilities

In this study, the proposed PCF-based THz waveguide is a structure comprised of several rectangular-shaped air holes. So far, many fabrication methods are available to fabricate PCF models. Among them, sol-gel casting [28], 3D-printed dies [29], extrusion [30], drawing [31], stacking, etc. are more familiar. A hollow-core PCF was fabricated using the 3D-printing method [32]. The fabricated waveguide showed a lower loss of about 0.02 cm^−1^ in the THz band. Another PCF-based THz waveguide was fabricated using polymer-jetting rapid prototyping [33]. This fabricated model attained quite similar optical parameters compared to its simulation results. As our proposed PCF model includes rectangular holes in both the core and cladding regions, it is not feasible to fabricate this model employing the sol-gel or stacking method. In the case of asymmetric microstructure fiber fabrication, the use of the extrusion method is more feasible [34]. In 2009, Atakaramians et al. [35] fabricate rectangular and spider-web shaped fibers by Polymethyl methacrylate (PMMA) polymer using the extrusion technique. The key reason behind the modelling of rectangle-based THz waveguide is the feasibility of heterodyne detection guaranteed by the rectangles [36,37].A similar type of rectangle-base PCF has recently been fabricated by the Max Planck Institute [29,38] which guarantees the feasible fabrication of our proposed rectangular-core PCF waveguide using the existing fabrication technique.

## 4. Numerical and Mathematical Methods

Dispersion is one of the significant barriers to multichannel communication. For this reason, our principal objective is to minimize the amount of dispersion of the proposed PCF model. Dispersion can occur due to background material used for fabrication. Another dispersion is waveguide dispersion, which occurs mainly through refraction while sending data through the core region. However, as we discuss before, Zeonex shows minimal dispersion variation in the THz band so that material dispersion will be negligible. In this study, we have calculated the waveguide dispersion using the following Equation (1) [25].

(1) $$\beta_{2}=\frac{2}{c}\frac{dn_{eff}}{d\omega }+\frac{\omega }{c}\frac{d^{2}n_{eff}}{d\omega^{2}}\;,\;\mathrm{ps}/\mathrm{THz}/\mathrm{cm}$$

where *n_eff_* represents the proposed fiber’s effective RI, ω is the angular frequency, and *c* indicates the free-space light velocity.

Another significant optical parameter of the PCF is birefringence. It is the difference between two effective RIs of the *x* and *y*-polarization modes of the fiber. It is calculated by the following Equation (2) [39].

(2) $$B=\left|n_{x}-n_{y}\right|$$

where *B* specifies the birefringence, *n_x_* and *n_y_* represent the RIs of the *x* and *y*-orthogonal polarization modes. The polarization property of PCF is measured based on birefringence. The higher birefringence ensures the effective polarization-maintaining applications of the PCF. 

The propagation constant, *β*, is introduced when a signal propagates through the core of a fiber. The value for this parameter, comprised of the real and imaginary part, varies as a function of propagating light frequency. The imaginary part of this parameter introduces leakage loss in the PCF model. The value for the imaginary part of this parameter, *β_i_*, is evaluated using the following Equation (3) [40,41].

(3) βi=Im[k×ηeff]=Im[2πfc×ηeff]=2πfc×Im[ηeff],  cm−1

where *k* is the wavenumber, *f* is the operating frequency, *η_eff_* is the effective refractive index, and Im[.] indicates the imaginary function.

Two-loss mechanisms, namely the EML and CL, are essential for every PCF-based waveguide. The EML mainly occurs because of the background material used in the PCF design. To reduce the EML, the use of porous core may be a solution, as high porosity minimizes the amount of background material [36]. The EML for our proposed PCF is evaluated using the following Equation (4) [42].

(4) αeff=(ε0μ0)12∫matnmat|E|2αmat dA2∫allSz dA ,cm−1

Here, *α_eff_* indicates the EML, *ε_0,_* and *µ_0_* denote the free-space permittivity and permeability individually, *n_mat_* is the RI of Zeonex, *E* defines the modal electric field, *α_nat_* defines the material absorption loss of Zeonex, and *S_z_* represents the *z*-component of Poynting vector.

The CL is another vital loss mechanism that can bound the length of signal transmission of the proposed PCF waveguide. The CL happens because of the inadequate structure of the cladding area of PCF, and the expansion of air holes in this area can minimize the CL [43]. It can be expressed by the following Equation (5) [2,44].

(5) αcl=(4πfc)Im(neff) , cm−1

where *α_cl_* represents the CL of the proposed PCF, Im(*n_eff_*) indicates the imaginary part of the effective RI, and *f* stands for the operating frequency. 

Another significant optical parameter of any PCF is sensitivity. It is the measure of the interaction between the total amount of passing light or data and material through the core. This parameter numerically defines the ability of any PCF to detect any specific analytes injected into the core region. The sensitivity is estimated using the Equation (6) [44,45,46].

(6) $$R=\frac{n_{mat}}{n_{eff}}\times P_{f}\%$$

where *n_mat_* is the RI of the material at the core holes, *n_eff_* indicates the effective RI of proposed fiber, and *P_f_* represents the core power fraction that is evaluated using the Equation (7) [45].

(7) $$P_{f}=\frac{\int_{Sample}^{}R_{e}\left(E_{x}H_{y}-E_{y}H_{X}\right)dxdy}{\int_{Total}^{}R_{e}\left(E_{x}H_{y}-E_{y}H_{x}\right)dxdy}\times 100$$

Here, the integration of the numerator in Equation (6) indicates the light power carried by the core hole material, and the integration of the denominator shows the total light power inside the fiber [1].

In the case of multichannel communication, the effective area is a significant property of a PCF. The region enclosed by the mode field within the core region of the PCF is known as the effective area, which is estimated by Equation (8) [47].

(8) $$A_{eff}=\frac{{\left[\int I\left(r\right)rdr\right]}^{2}}{\left[\int I^{2}\left(r\right)dr\right]}$$

where *I*(*r*) = |*E_t_*|^2^ represents the electric field intensity across the PCF waveguide.

In the core area of a PCF, the quantity of gathered power can be realized by the numerical aperture, and it relies on the effective area of the proposed PCF [48]. The numerical aperture (*N_A_*) of the proposed waveguide is calculated using the Equation (9) [1]:

(9) $$N_{A}=\frac{1}{\sqrt{1+\frac{\pi A_{eff}}{\lambda^{2}}}}$$

where *λ* is the wavelength of the operating signal.

## 5. Results and Discussions

The performance of the proposed model has been examined twice. Firstly, the optimum 48 R model has been studied and compared with the 24 R and 80 R model. Then the porosity of the 48 R model has been tuned to find the optimum porosity. Both the optical parameters for both these studies have been presented in Section 5.1 and Section 5.2.

### 5.1. Optimum Model Selection

**Figure 3:**
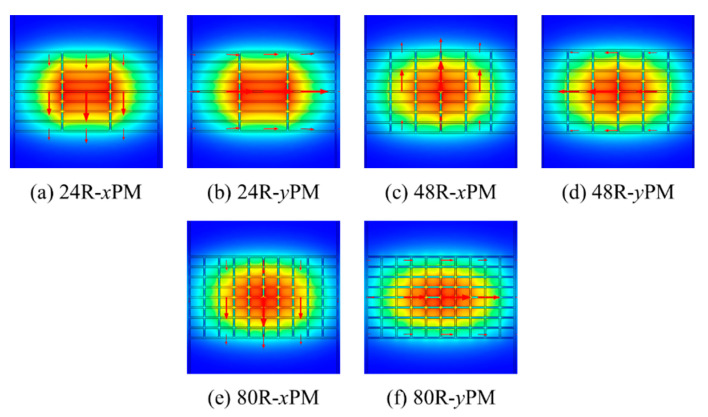
THz wave propagation profile for the 24 R, 48 R, and 80 R model.

To justify the superiority of the 48 R model, this model has been compared with the 24 R and 80 R model. The strut value for all these three models is the same. The THz wave propagation profile through the core of the 24 R, 48 R, and 80 R model in the *x*-polarization mode (PM) and *y*-PM is shown in Figure 3.

**Figure 4:**
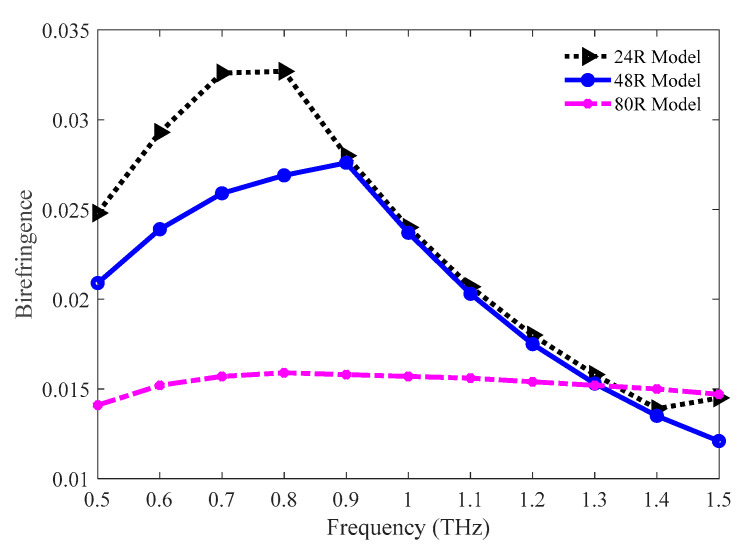
Comparison of birefringence among the 24 R, 48 R, and 80 R model.

The values of birefringence for the 24 R, 48 R, and 80 R models have been compared and presented in Figure 4. The value of birefringence is higher for the 24 R model at lower frequencies. But all the models show adjacent values for birefringence at higher frequencies. The values of birefringence for the 24 R, 48 R, and 80 R models are 0.0158, 0.0153, and 0.0152, respectively, at 1.3 THz.

**Figure 5:**
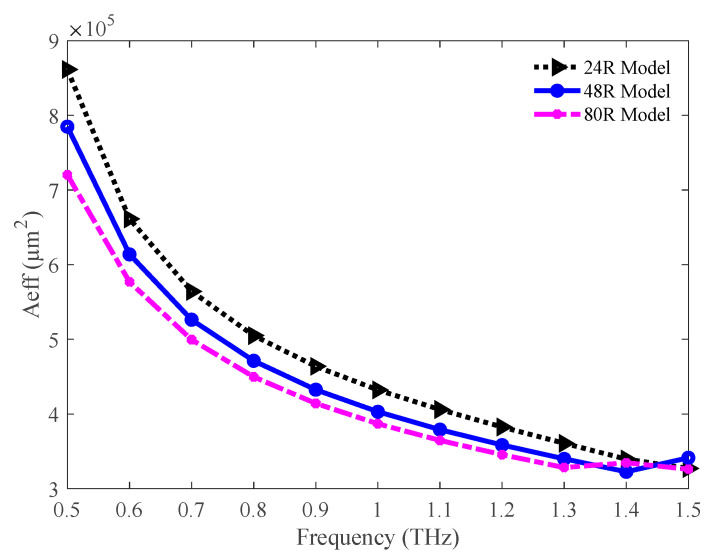
Depiction of effective areas for the 24 R, 48 R, and 80 R model.

The comparison of the effective areas among the three variants of the proposed model is shown in Figure 5. The effective area decreases with increasing frequency for all three variants. This indicates the presence of high light power inside the core. The values of the effective area are 36.08 × 10^4^, 34.00 × 10^4^, and 32.84 × 10^4^ µm^2^ for the 24 R, 48 R, and 80 R models, respectively, at 1.3 THz.

**Figure 6:**
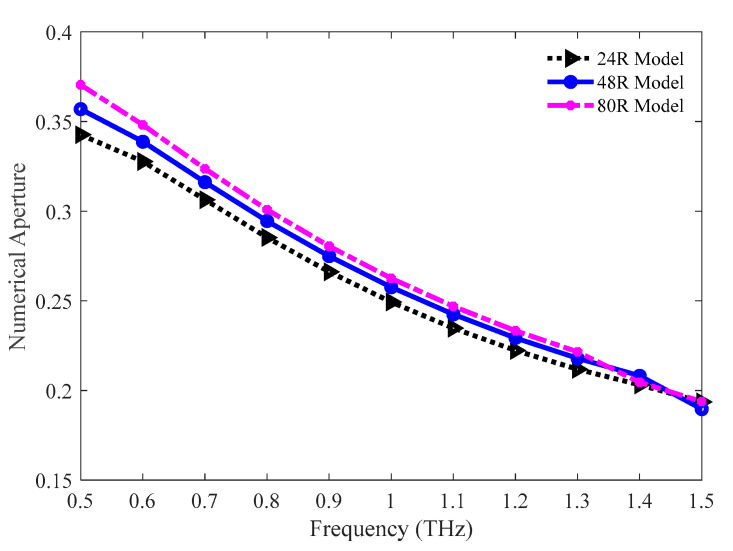
Representation of the numerical aperture for the three variants of the optimum model.

The comparison of the numerical apertures among the 24 R, 48 R, and 80 R model is pictured in Figure 6. As the value of the numerical aperture is a function of both the effective area and the operation frequency, the values of it also decrease with the increasing frequency following the slopes of the effective areas. The values of numerical aperture are 0.2118, 0.2179, and 0.2216 for the 24 R, 48 R, and 80 R models individually at 1.3 THz.

**Figure 7:**
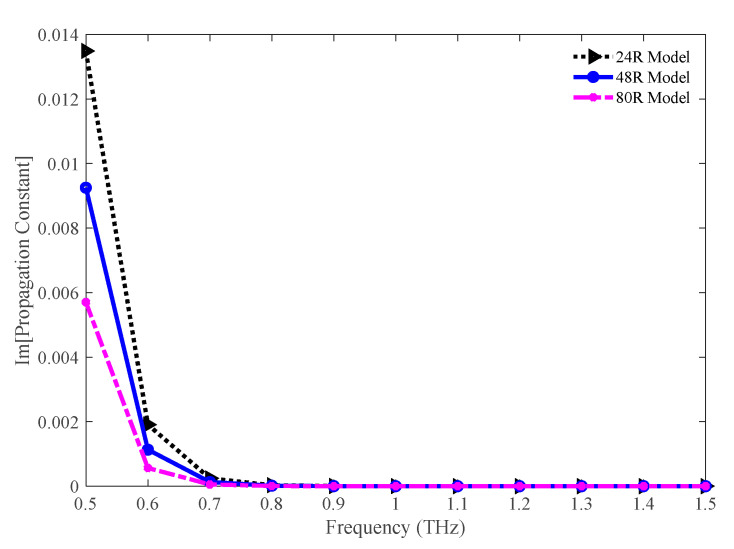
Comparison of the imaginary part of the propagation constant among the 24 R, 48 R, and 80 R model.

Figure 7 depicts the imaginary part of the propagation constant for the 24 R, 48 R, and 80 R model. Though the values for this parameter are higher initially, the values decrease with an increase in the operating frequency. As this parameter introduces leakage losses, lower values of it are desirable. The values of this parameter for 24 R, 48 R, and 80 R model are 8.66 × 10^−10^, 2.89 × 10^−10^, 6.24 × 10^−11^ cm^−1^, respectively, at 1.3 THz.

**Figure 8:**
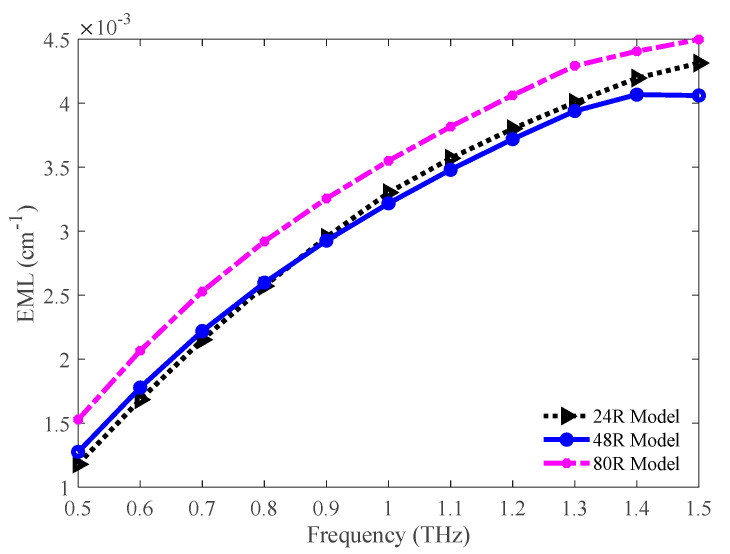
Comparison of the EML among the 24 R, 48 R, and 80 R model.

The values of EML for the 24 R, 48 R, and 80 R models have been graphically compared in Figure 8. The EML increases with the increasing frequency, which indicates higher light absorption by the bulk material at a higher frequency. The 80 R model exhibits a higher EML. However, the lowest EML is attained for the 48 R model. The values of EML for the 24 R, 48 R, and 80 R model are 0.004, 0.0039, and 0.0043 cm^−1^ respectively at 1.3 THz.

**Figure 9:**
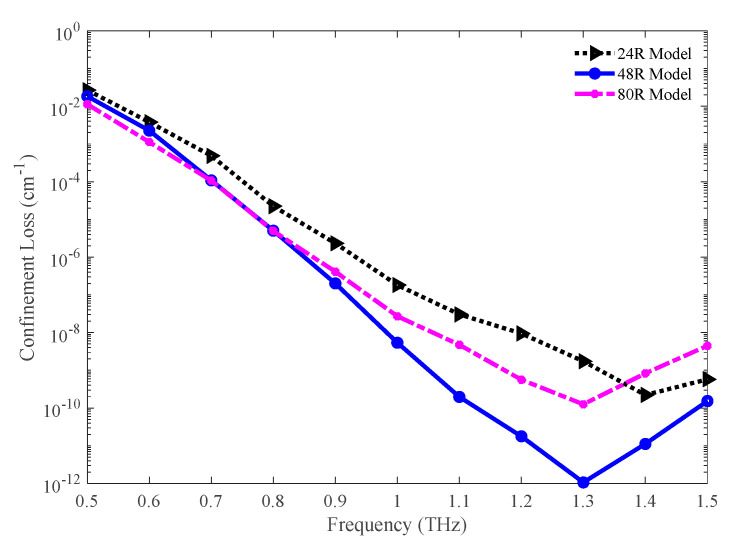
Representation of the confinement loss for the 24 R, 48 R, and 80 R model. Note that, Matlab function *semilogy* () has been used for better visualization of the difference among three models.

The confinement losses for the three variants of the optimum model are presented in Figure 9. Initially, the confinement loss is higher for all three models. But, the values of confinement loss are lower for higher frequency. This is also an indication of higher light confinement at a higher frequency. The values of this loss are 1.73 × 10^−9^, 1.06 × 10^−12^, and 1.25 × 10^−10^ cm^−1^ for the 24 R, 48 R, and 80 R model correspondingly at 1.3 THz.

**Figure 10:**
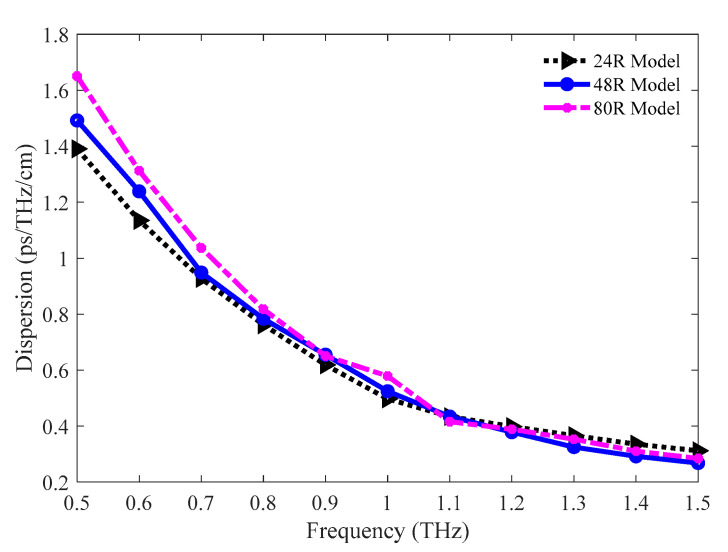
Comparison of dispersion among the 24 R, 48 R, and 80 R model.

One of the vital optical parameters of any waveguide that needs to be examined is the dispersion. This parameter limits the volume of the carried signal of any PCF. The value of it depends on both the waveguide and the bulk material and the use of Zeonex in the proposed model eliminates the effect of material dispersion. The waveguide dispersions for the 24 R, 48 R, and 80 R models have been studied and presented in Figure 10. Nearly flat dispersion is found for the three models in the frequency band ranging from 1.2 to 1.5 THz. Moreover, all these models show an ultra-lower amount of dispersion at this band since the lowest amount of dispersion noticed in the THz waveguides discussed in Section 1 is 0.38 ps/THz/cm. The values of dispersion for the 24 R, 48 R, and 80 R models are 0.3659, 0.3251, and 0.3529 ps/THz/cm individually at 1.3 THz.

**Figure 11:**
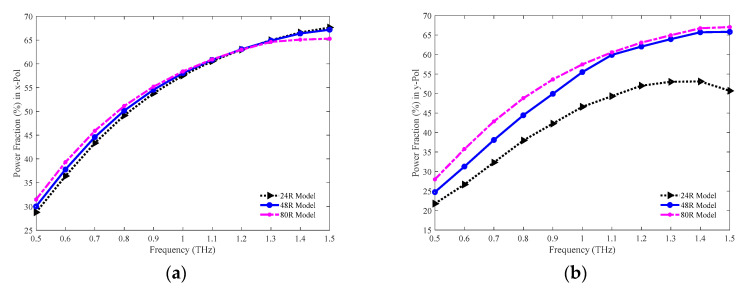
Comparison of the power fraction among the 24 R, 48 R, and 80 R model in the (**a**) *x* and (**b**) *y*-PM.

The comparison of power fraction in both the *x* and *y*-PM among the 24 R, 48 R, and 80 R models are presented in Figure 11a,b individually. At higher frequency, the fraction of light power present at the core increases for the three models in both cases. The values of power fraction in the *x*-PM are 64.98, 64.90, and 64.63% for the 24 R, 48 R, and 80 R model correspondingly at 1.3 THz. Whereas in the *y*-PM, the values of power fraction are 53.02, 63.94, and 64.94% respectively for the 24 R, 48 R, and 80 R model individually at the same frequency.

The values of the optical parameters for the 24 R, 48 R, and 80 R model are shown in Table 1. No particular model shows dominant values for all the optical parameters, for instance, the highest power fraction in the *x*-PM is found for the 24 R model, but it has the lowest value in the *y*-PM. However, the 48 R model shows the lowest values for the confinement loss, EML, and dispersion. Besides, this model maintains standard values for all the parameters. Hence the 48 R model has been elected as the optimum model for this study. Besides, we have chosen 1.3 THz as the operating frequency for this waveguide because flat dispersion, high power fraction, moderately lower EML, and lower confinement loss are present at this point, as depicted in Table 1.

### 5.2. Optimum Porosity Selection

**Figure 12:**
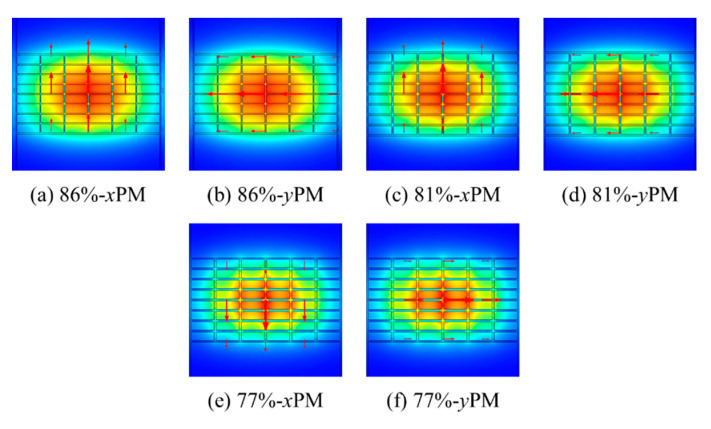
THz wave propagation profile for the 86%, 81%, and 77% porosity model.

To realize the optimum porosity for the 48 R model, we have tuned the strut value in the core region which results in three different values of porosity, i.e., 86% (for 7.5 µm strut), 81% (for 10 µm strut), and 77% (for 12.5 µm strut). The values for the optical parameters for these three porosities have been evaluated. The THz wave propagation profile through the core of the 86%, 81%, and 77% porosity model in the *x*-PM and *y*-PM is shown in Figure 12.

**Figure 13:**
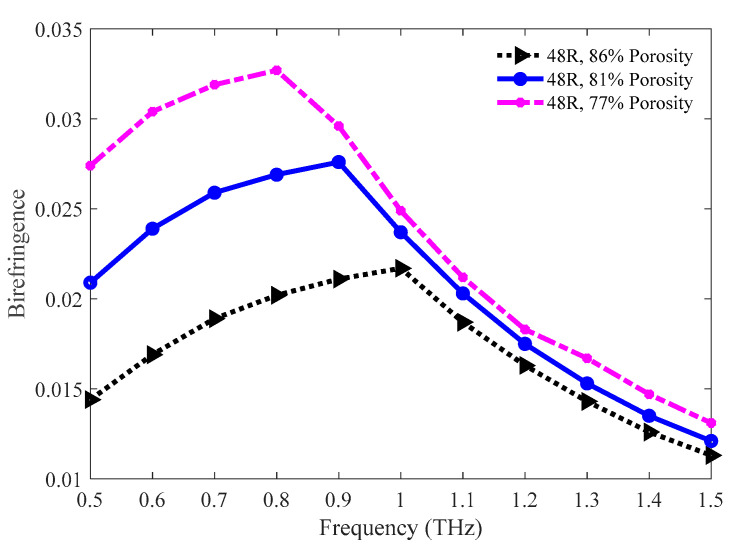
Comparison of the birefringence among the 86%, 81%, and 77% porosity model.

Figure 13 pictures the comparison of birefringence among the 86%, 81%, and 77% porosity of the 48 R model. Both the 77% and 81% porosity model show higher birefringence throughout the operating region. The values of birefringence for 86%, 81%, and 77% porosity model are 0.0143, 0.0153, and 0.0167, respectively, at 1.3 THz.

**Figure 14:**
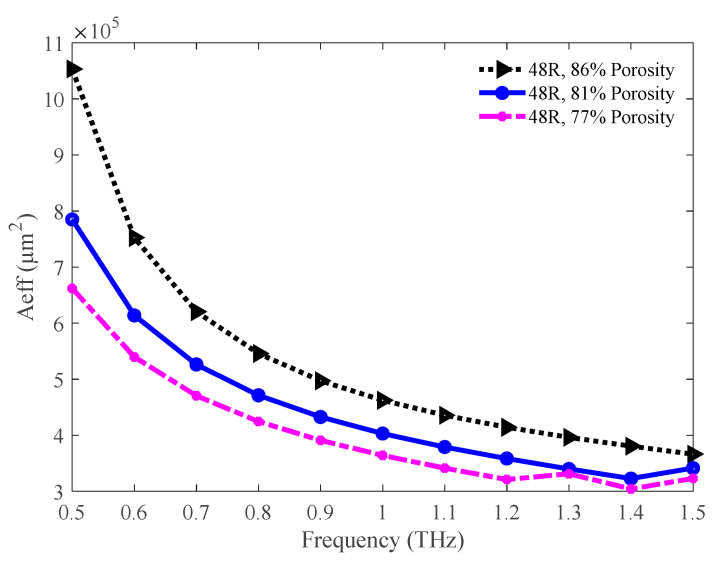
Comparison of effective areas among the 86%, 81%, and 77% porosity model.

The values of the effective area in the THz band for the three different porosity of the 48 R model is presented in Figure 14. For all three cases, the effective area decreases with an increase in the operating frequency. Since the value of the effective area is lower for lower porosity, light spreading is lower for this model. The values of the effective area are 39.63 × 10^4^, 34.00 × 10^4^, and 33.13 × 10^4^ µm^2^ for the 86%, 81%, and 77% porosity model correspondingly at 1.3 THz.

**Figure 15:**
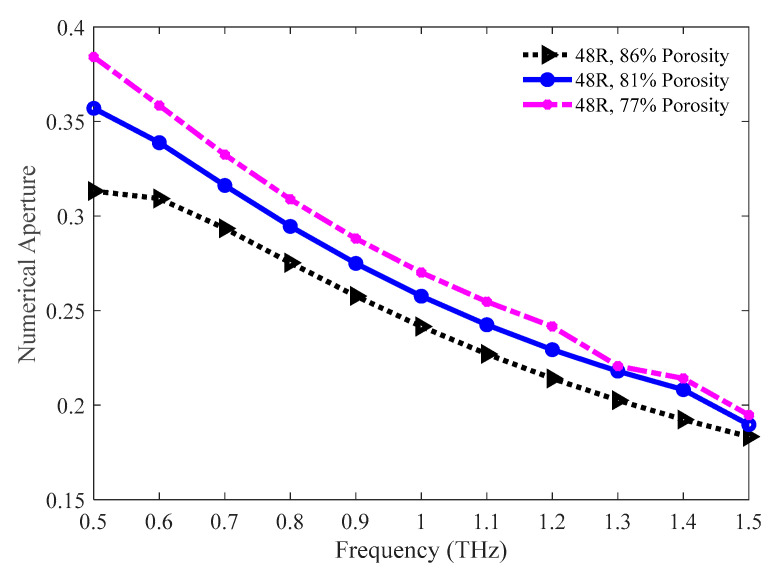
The fluctuation of the numerical aperture for the 86%, 81%, and 77% porosity model in the frequency band ranging from 0.5 to 1.5 THz.

The values of the numerical aperture for the 86%, 81%, and 77% porosity model are graphically compared in Figure 15. A higher numerical aperture is attained for the lower porosity model as the effective area is lower for this model. However, 81% and 77% model show nearly similar numerical aperture at 1.3 THz. The values of numerical aperture are 0.2025, 0.2179, and 0.22 for the 86%, 81%, and 77% porosity model individually at 1.3 THz.

**Figure 16:**
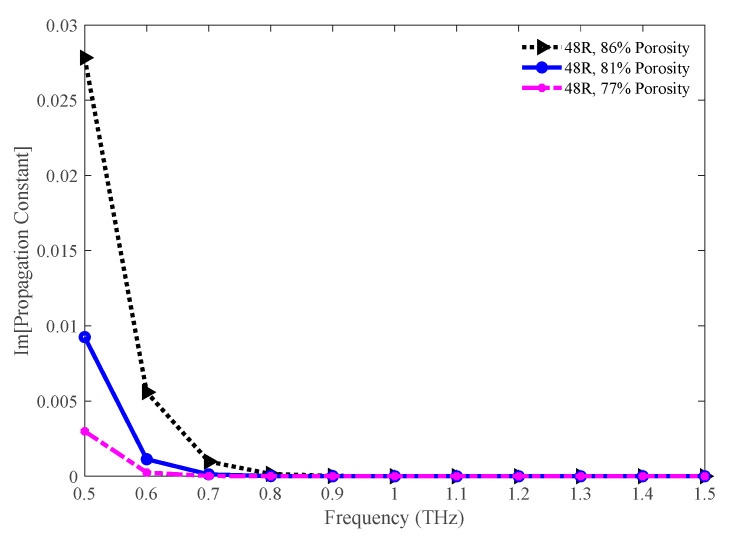
Comparison of the imaginary part of the propagation constant among the 86%, 81%, and 77% porosity model.

Figure 16 represents the imaginary portion of the propagation constant for the 86%, 81%, and 77% porosity model. The value for this parameter is higher at a lower frequency. But it becomes significantly low at a higher frequency. At 1.3 THz, the value of this parameter for the 86%, 81%, and 77% porosity model are 1.91 × 10^−8^, 2.90 × 10^−10^, and 1.60 × 10^−10^ cm^−1^, respectively. The values of this parameter imply that the leakage loss is quite insignificant for our proposed model.

**Figure 17:**
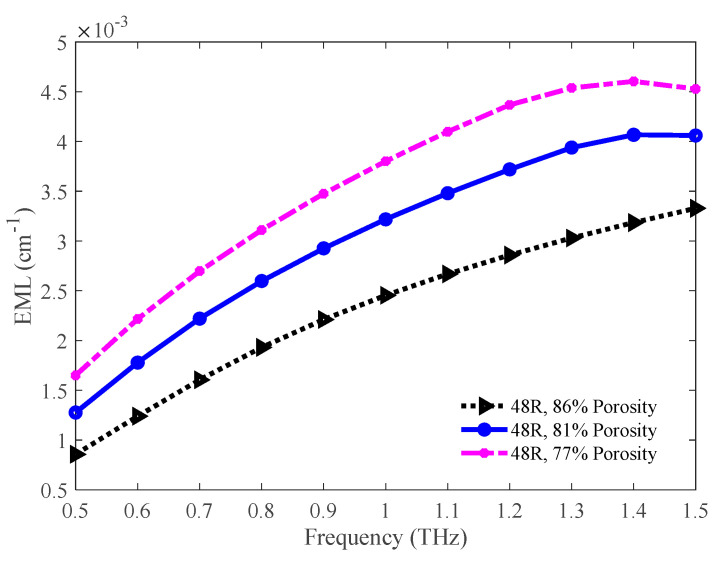
Representation of the EML for the 86%, 81%, and 77% porosity model.

The comparison of EML among the 86%, 81%, and 77% porosity model is shown in Figure 17. Opposite to the effective area, the EML is higher for the lower porosity model. This is due to the presence of a higher volume of background material in the lower porosity model. The values of EML for the 86%, 81%, and 77% porosity model are 0.0030, 0.0039, and 0.0045 cm^−1^ exclusively at 1.3 THz.

**Figure 18:**
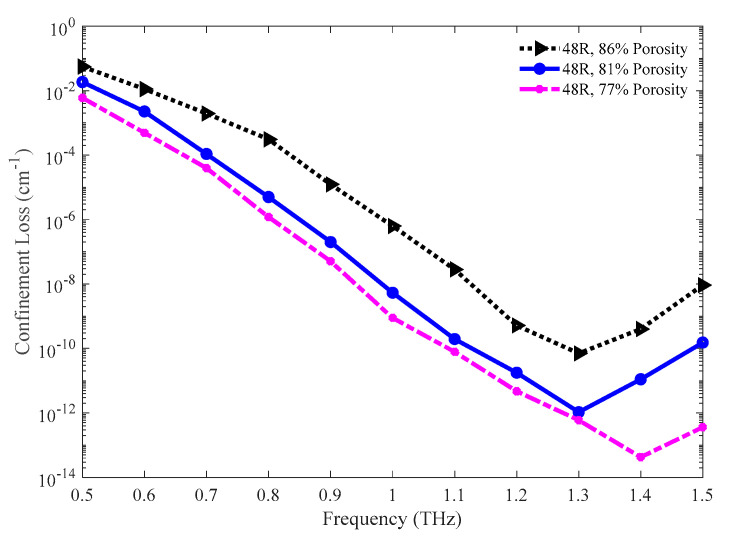
Comparison of confinement loss among the 86%, 81%, and 77% porosity model.

The confinement losses for the three porosity models are shown in Figure 18. The lower confinement loss is found for lower porosity. However, all three models exhibit a very lower amount of confinement loss immediately after 0.8 THz. The values of confinement loss for the 86%, 81%, and 77% porosity model are 7.03 × 10^−11^, 1.06 × 10^−12^, and 5.96 × 10^−13^ cm^−1^ individually at 1.3 THz.

**Figure 19:**
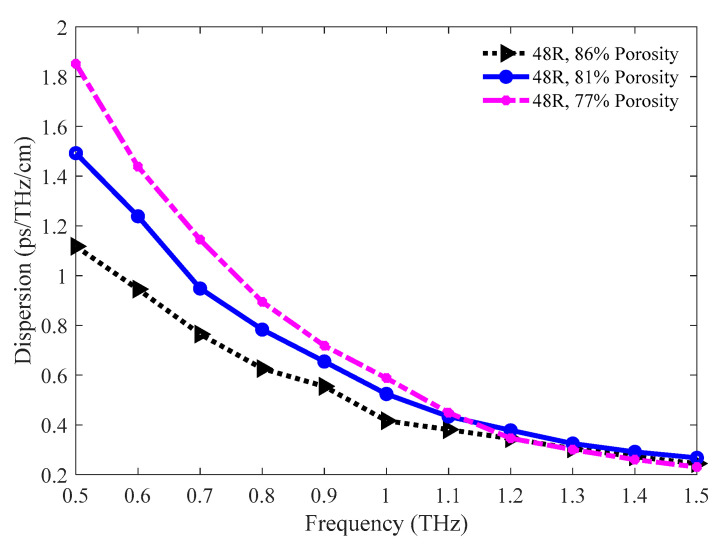
Representation of dispersion for the 86%, 81%, and 77% porosity model.

The amounts of dispersion for the 86%, 81%, and 77% porosity model are pictured in Figure 19. All three models show lower waveguide dispersion in the operating region. Nearly flat dispersion is found in the 1.2 to 1.5 THz band. The amounts of dispersion for the 86%, 81%, and 77% porosity model are 0.3045, 0.3251, and 0.3004 ps/THz/cm correspondingly at 1.3 THz.

**Figure 20:**
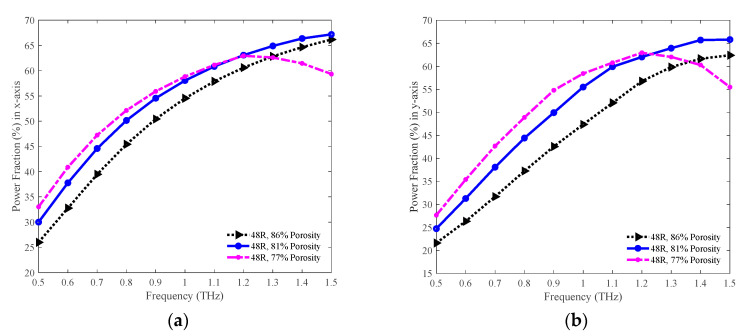
Representation of the power fraction for the 86%, 81%, and 77% porosity model in the (**a**) *x* and (**b**) *y*-PM.

The values of power fraction in both the *x* and *y*-PM for the 86%, 81%, and 77% porosity model are shown in Figure 20a,b separately. In both PM, initially, the power fraction is higher for the 77% porosity model, but it becomes lower compared to the other models at higher frequencies. The highest power fraction attained in both PM is for the 81% porosity model at 1.3 THz. The values of power fraction in the *x*-PM are 62.84, 64.90, and 62.60% for the 86%, 81%, and 77% porosity model individually at 1.3 THz. In contrast, in the *y*-PM, the amounts of power fraction are 59.84, 63.94, and 62.04% respectively for the 86%, 81%, and 77% porosity model individually at the same frequency.

The values of the optical parameters for the 86%, 81%, and 77% porosity model are shown in Table 2. No particular porosity model shows dominant values for all the optical parameters; for instance, the lowest EML is attained for the 86% porosity model. Still, the lowest confinement loss is found for the 77% porosity model. The 81% porosity is selected as the optimum porosity because this model shows dominant values for the power fraction. Further, this model maintains standard values for the other optical parameters. Hence the 48 R model with 81% porosity is the proposed PCF model for this study. This model has a strut value of 10 µm. The minimum acceptable strut value is 6.5 µm, so 10 µm strut will provide fabrication feasibility [28,49].

**Figure 21:**
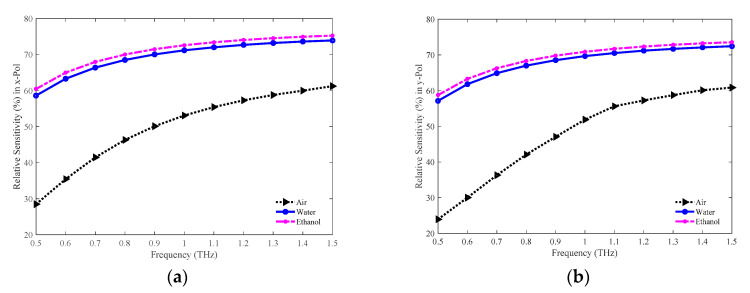
Comparison of the relative sensitivities among the air, water, and ethanol in the (**a**) *x* and (**b**) *y*-PM.

The relative sensitivities in the *x* and *y*-PM for the optimum model (48 R–81% porosity model) in detecting air, water, and ethanol are shown in Figure 21a,b separately. Higher sensitivities are attained for higher refractive indexed analytes (air < water < ethanol). The highest sensitivity is achieved for the ethanol in both PMs. The values of sensitivity in the *x*-PM for the air, water, and ethanol are 58.79%, 73.20%, and 74.55% exclusively at 1.3 THz. Whereas, in the *y*-PM, the sensitivity is 58.73%, 71.70%, and 72.85% the air, water, and ethanol respectively, at the same frequency. Figure 21 implies that the modelled PCF can also be applied in gas, chemical, and bio-sensing applications.

A comparison of our proposed PCF-based THz waveguide with the existing THz waveguides is presented in Table 3. The proposed model has a very low level of dispersion. Compared to the existing models presented in Table 3, the proposed model has the lowest dispersion. The effective area for the proposed model is very high compared to the other models. The EML for our proposed model is the lowest compared to the other models. The confinement loss is also lower. Moreover, the power fraction for the proposed model has the highest value compared to the other models presented in Table 3.

## 6. Conclusions

This study designs an ultra-lower loss and ultra-lower dispersion PCF model for the THz propagation. Several optical parameters have been studied to examine the efficiency of the proposed model. This efficiency evaluation is accomplished in the THz band that ranges from 0.5 to 1.5 THz. We have compared the proposed model with dissimilar structured models as well as with varied porosity models. All the results represent the higher efficiency of the proposed PCF model in THz wave propagation. The proposed model shows dominant results compared to the other models. 

The proposed model shows dominant values for multiple optical parameters compared to the others. This model has the highest power fraction of about 65%. Moreover, the dispersion is the lowest for this model compared to the others. Also, this model has the lowest dispersion and lower confinement loss. All these values demonstrate the superiority of this model. However, the use of rectangles to model the proposed PCF structure ensures the fabrication possibilities. Hence, this proposed PCF would be a promising applicant in the field of biomedical, telecommunication, biosensing, and chemical sensing which is now under investigation.

## Figures and Tables

**Table 1:** Values of optical parameters for the 24 R, 48 R, and 80 R model at 1.3 THz.

Model Type	Bir	Eff. Area (μm^2^)	NA	EML (cm^−1^)	Conf. Loss (cm^−1^)	Dispersion (ps/THz/cm)	PF in *x*-Pol (%)	PF in *y*-Pol (%)
24 R	0.0158	36.08 × 10^4^	0.212	0.0040	1.73 × 10^−9^	0.3659	64.98	53.02
**48 R**	**0.0153**	**34.00 × 10^4^**	**0.218**	**0.0039**	**1.06 × 10^−12^**	**0.3251**	**64.90**	**63.94**
80 R	0.0152	32.84 × 10^4^	0.221	0.0043	1.25 × 10^−10^	0.3529	64.63	64.94

**Table 2:** Values of optical parameters for the 86%, 81%, and 77% porosity model at 1.3 THz.

Model Porosity	Bir	Eff. Area (μm^2^)	NA	EML (cm^−1^)	Conf. Loss (cm^−1^)	Dispersion (ps/THz/cm)	PF in *x*-Pol (%)	PF in *y*-Pol (%)
86%	0.0143	3963 × 10^4^	0.202	0.003	7.03 × 10^−11^	0.3045	62.84	59.84
**81%**	**0.0153**	**34.00 × 10^4^**	**0.218**	**0.0039**	**1.06 × 10^−12^**	**0.3251**	**64.90**	**63.94**
77%	0.0167	3313 × 10^4^	0.22	0.0045	5.96 × 10^−13^	0.3004	62.60	62.04

**Table 3:** Comparison of our proposed THz waveguide with previously proposed THz waveguides.

Year [Ref.]	Frequency (THz)	Dispersion (ps/THz/cm)	Bir	Eff. Area (μm^2^)	NA	EML (cm^−1^)	Conf. Loss (cm^−1^)	PF (%)
2015 [18]	1.0	0.5	0.075	―	―	0.12	0.069	22.00
2016 [19]	1.0	1.42	0.048	―	―	0.12	5.16 × 10^−6^	31.00
2016 [20]	1.0	0.9	0.045	―	―	0.08	―	33.00
2016 [22]	3.2	0.51	0.03	―	―	0.6	2.3 × 10^−5^	46.00
2016 [21]	0.7	2.92	0.0105	2.3 × 10^5^	―	0.076	0.576	―
2017 [23]	1.0	―	0.012	―	―	0.0689	―	―
2017 [50]	0.7	0.38	―	―	―	0.0118	1.0	17.00
2017 [25]	1.0	0.5	0.063	1.24 × 10^5^	―	0.081	1.96 × 10^−3^	46.90
2018 [51]	1.1	1.35	0.063	1.2 × 10^5^	―	0.06	5.45 × 10^−13^	45.00
2019 [52]	1.0	4.0	―	1.1 × 10^5^	0.45	0.1	1.38 × 10^−12^	57.50
2019 [53]	1.2	1.4	0.096	4.7 × 10^4^	―	0.055	5.0 × 10^−5^	28.67
**2020 [Proposed]**	**1.3**	**0.3251**	**0.0153**	**3.4 × 10^5^**	**0.22**	**0.0039**	**1.06 × 10^−12^**	**64.90**
